# The coding-complete genome sequence of Arabidopsis latent virus 1 from Iraq

**DOI:** 10.1128/mra.00703-26

**Published:** 2026-08-04

**Authors:** Athraa A. Hadi, Mushtak T. Mohammadali, Sienaa M. Al-Zurfi, Adnan A. Lahuf

**Affiliations:** 1 Plant Protection Department, College of Agriculture, University of Kerbala 125663, Karbala, Iraq; Portland State University, Portland, Oregon, USA

**Keywords:** Arabidopsis latent virus 1, *Comovirus*, *Secoviridae*, complete genome, Iraq

## Abstract

Here, we report the coding-complete genome sequence of Arabidopsis latent virus 1 (ArLV1; *Comovirus arabidopsis*) from Iraq. The virus was identified by metatranscriptomic sequencing of asymptomatic cucumber (*Cucumis sativus*) leaves harboring thrips collected from commercial greenhouses. The bipartite genome comprises RNA1 (5,553 nt) and RNA2 (3,582 nt).

## ANNOUNCEMENT

Arabidopsis latent virus 1 (ArLV1; *Comovirus arabidopsis*), a member of the family *Secoviridae*, genus *Comovirus*, possesses a bipartite, positive-sense, single-stranded RNA genome (1). The virus is widely distributed in *Arabidopsis thaliana* collections worldwide, typically establishing persistent, asymptomatic infections (2). Several viruses infecting cucurbit crops have been reported in Iraq (3, 4).

In April 2025, asymptomatic cucumber (*Cucumis sativus*) leaves (20 leaf samples) harboring *Thrips* spp. insects were collected from commercial plastic greenhouses in northern and central Iraq. Pooled leaf and insect material was submitted to BGI (Hong Kong, China) for metatranscriptomic sequencing. Total RNA was extracted from pooled samples and treated with DNase I to eliminate DNA contaminants (5). Ribosomal RNA was depleted using the Ribo-off rRNA Depletion Kit (MGI Tech), followed by RNA fragmentation. First- and second-strand cDNA synthesis (6) was performed using random hexamer primers, with dUTP incorporated in place of dTTP during second-strand synthesis. The resulting cDNA fragments were end-repaired (7), A-tailed, adaptor-ligated, and PCR-amplified. After library quality control (8), products were denatured, cyclized, converted into DNA nanoballs (DNBs), and sequenced on the DNBSEQ platform (BGI, Hong Kong) (9). A total of 36,126,439 raw reads were quality-trimmed using Trimmomatic v0.32. Host reads were removed by mapping against the *C. sativus* reference genome (GCF_000004075.3) with Bowtie2 v2.5.5; the remaining 152,574 non-host reads were *de novo* assembled with Velvet v1.2.10 (10), producing 33,631 contigs. Resulting contigs were screened by BLASTn (11) against National Center for Biotechnology Information (NCBI) plant virus sequences, and viral contigs were validated and characterized by reference-based assembly in Geneious Prime 2024.0.4 (Biomatters Ltd., New Zealand) using ArLV1 reference sequences NC_078590.1 (RNA1) and NC_078591.1 (RNA2).

Two contigs matched both ArLV1 genome segments with >98–99% pairwise nucleotide identity and 100% query coverage with sequence depths of 28× and 16×, respectively. The coding-complete genome of the Iraqi isolate (ArLV1-Iraq1) consists of RNA1 (5,553 nt; guanine-cytosine [GC] content 42.0%) encoding the RNA-dependent RNA polymerase and RNA2 (3,582 nt; GC content 41.9%) encoding the movement protein–coat protein polyprotein with 100% segment coverage. The highest nucleotide identities were shared with a Netherlands isolate (RNA1: 99.75%, GenBank accession PQ342002.1; RNA2: 99.55%, NC_078591.1) (Table 1). To our knowledge, this represents the first coding-complete genome sequence of ArLV1 reported from Iraq.

**TABLE 1:** Pairwise nucleotide sequence identity between the Iraqi isolate ArLV1-Iraq1 and its closest global isolates (11)

The virus	Closest isolates	% identity	Year of collection	Country
Arabidopsis latent virus 1 RNA 1	PQ342002.1	99.75%	2024	Netherlands
	NC_078590.1	99.56%	2023	Netherlands
	MH899120.1	99.21%	2019	Netherlands
Arabidopsis latent virus 1 RNA 2	NC_078591.1	99.55%	2023	Netherlands
	PQ342003.1	99.42%	2024	Netherlands
	MH899121.1	99.11%	2019	Netherlands

## Data Availability

The coding-complete genome sequences of ArLV1-Iraq1 have been deposited in GenBank under accession numbers PZ167518.1 (RNA1) and PZ167519.1 (RNA2). The associated BioProject and BioSample accession numbers are PRJNA1440257 and SAMN56608350, respectively. The raw sequencing data are deposited in the NCBI SRA under accession number SRR38469432. All records are accessible at https://www.ncbi.nlm.nih.gov.
